# Response Parameters for SMS Text Message Assessments Among Pregnant and General Smokers Participating in SMS Cessation Trials

**DOI:** 10.1093/ntr/ntv266

**Published:** 2015-12-12

**Authors:** Felix Naughton, Muhammad Riaz, Stephen Sutton

**Affiliations:** Behavioural Science Group, University of Cambridge, Cambridge, United Kingdom

## Abstract

**Introduction:**

Despite a substantial increase in use of SMS text messages for collecting smoking-related data, there is limited knowledge on the parameters of response. This study assessed response rates, response speed, impact of reminders and predictors of response to text message assessments among smokers.

**Methods:**

Data were from two SMS cessation intervention trials using clinical samples of pregnant (*n* = 198) and general smokers (*n* = 293) sent text message assessments during 3-month cessation programs. Response rates were calculated using data from the host web-server. Changes in response over time, impact of reminders and potential demographic (age, gender, ethnicity, parity, and deprivation) and smoking (nicotine dependence, determination to quit, prenatal smoking history, smoking status at follow-up) predictors of response were analyzed.

**Results:**

Mean response rates were 61.9% (pregnant) and 67.8% (general) with aggregated median response times of 0.35 (pregnant) and 0.64 (general) hours. Response rate reduced over time (*P* = .003) for general smokers only. Text message reminders had a significant effect on response (*P*s < .001), with observed mean increases of 13.8% (pregnant) and 17.7% (general). Age (odds ratio [*OR*] = 0.95, 95% confidence interval [CI] 0.90–1.00) and deprivation (*OR* = 0.98, 95% CI 0.96–1.00) weakly predicted response among pregnant smokers and nonsmoking status at 4 weeks follow-up (*OR* = 8.63, 95% CI 3.03–24.58) predicted response among general smokers.

**Conclusions:**

Text message assessments within trial-based cessation programs yield rapid responses from a sizable proportion of smokers, which can be increased using text reminders. While few sources of nonresponse bias were identified for general smokers, older and more deprived pregnant women were less likely to respond.

**Implications:**

This study demonstrates that most pregnant and general smokers enrolled in a cessation trial will respond to a small number of questions about their smoking sent by text message, mostly within 1 hour of being sent the assessment text message. For those who do not initially respond, our findings suggest that 24- and 48-hour text message reminders are likely to increase response a small but meaningful amount. However, older age and higher deprivation among pregnant smokers and relapse among general smokers is likely to reduce the chance of response.

## Introduction

SMS text messages are recommended as a method for collecting data from participants of medical research^1^ and are increasingly used for doing so, including for smoking behavior.^2^ Text messages have high reach potential for data collection due to very high mobile phone ownership in developed nations and rapidly increasing ownership in developing nations.^3^ While smoking studies using SMS assessments often report response rates, they seldom explore time to response, and have not examined interventions to increase response or predictors of response.

Bridging these knowledge gaps is important to help determine the utility of text messaging for measuring smoking characteristics and to inform the design of digital smoking cessation interventions. For example, estimating the speed of response profile and the impact of reminders would help optimize the use and timing of reminders and help inform interventions contingent on data input during delivery such as tailored interventions.^4,5^ A record of lapses or relapse to smoking, changes in determinants of relapse or engagement in a period of abstinence can be used by eHealth and mHealth interventions to dynamically trigger support and tailor the type and intensity of that support.^6,7^ Increasing our knowledge of factors associated with response would help identify potential biases when text messages were used exclusively for assessment purposes and identify groups who might become underserved by interventions where effectiveness may be partially dependent on interaction.

This study uses data from two text message smoking cessation intervention trials. In addition to a general sample, a pregnant sample is included given this group’s distinct quitting motivations and public health priority status. Three research questions are addressed: (1) What are the response rates and speed of response to text message assessments sent to smokers participating in intervention studies? (2) What impact do text message assessment reminders have on response? (3) Which characteristics are associated with response?

## Methods

### Sample and Design

Data from two randomized controlled trials evaluating smoking cessation SMS text message interventions were used. Only participants in these trials who were sent text message assessments were included in the present study; participants from both control and intervention arms (*n* = 198) of a trial of pregnant smokers (MiQuit) and the intervention arm participants only (*n* = 293) of a trial of general (ie, nonpregnant) smokers (iQuit in Practice). Inclusion was restricted to trial participants sent an initial text message assessment enquiring about smoking status (3 weeks post-enrolment).

#### MiQuit Trial

Pregnant smokers were recruited via seven UK National Health Service (NHS) Trusts. Trial inclusion criteria were 16 years of age or over, under 21 weeks pregnant, smoked seven or more cigarettes per week, had regular use of a mobile phone and could understand written English.

#### iQuit in Practice Trial

Participants were patients receiving smoking cessation support at one of 32 UK General Practices. Trial inclusion criteria were aged 18–75 years, smoked at least one cigarette a day, had regular use of a mobile phone, willing to set a quit date within 14 days, and not using smoking cessation medications at randomization.

Participants received a £5 (iQuit) or £10 (MiQuit) shopping voucher for trial participation. Further details are reported elsewhere, including descriptions of the interventions, behavior change techniques used and support delivery schedules.^8,9^ Trial withdrawals were excluded from analysis (MiQuit *n* = 9, iQuit *n* = 2). NHS ethical approval was obtained for both trials (06/Q0108/301; 09/H0308/87).

### Procedure

Intervention arm participants in both trials received a 3-month program of automated smoking cessation support text messages plus a tailored advice leaflet/report. Intervention participants in both trials and control participants in the pregnant smokers trial (who received assessment texts only) were sent text message based assessments at multiple timepoints; 3, 5, and 7 weeks (MiQuit) and 3 and 7 weeks (iQuit) post-enrolment. The week 3 and 7 messages invited a reply to confirm whether participants had smoked within 7 days: (MiQuit) “Hi [name], have you been smoking in the last 7 days (even a puff)? Reply with YES if you have smoked or NO if you haven’t smoked”; (iQuit) “Hi [name], How’s it going? Have u smoked at all (even a puff) in the last week? If u have smoked reply YES now, otherwise text NO. Thanks.” The week 5 message (MiQuit only) assessed confidence in quitting on a 5-point scale: “Hi [name], how confident do you feel in quitting for the remainder of your pregnancy? Reply with a number from 1 to 5 (1 = not at all, 5 = extremely).” A participant reply triggered a response text. Assessment messages were sent at randomly selected times between 10.05–13.05 (MiQuit) and 10.05–15.05 (iQuit) on the day the message was scheduled to be sent. Reminders were sent if no reply was received within 24 hours, apart from the MiQuit 5- and 7-week assessments where reminders were sent after 48 hours. Reminder texts highlighted that a response had not yet been received and repeated the assessment question. Responses to these assessment text messages and reminders form the focus of the current study. No other text messages sent during the programmes requested a response.

### Measures

Response and time to response to text assessments were recorded by a University of Cambridge webserver. Response rates are defined as the proportion of participants who text a reply for each assessment text sent (three per MiQuit participant; two per iQuit participant). Potential predictors of response were: nicotine dependence using an adapted Heaviness of Smoking Index (excluding “≤5 minutes” category for time to first cigarette on waking),^10^ smoking status (MiQuit: cotinine-validated 2-week point prevalence abstinence 12 weeks post-enrolment; iQuit: Carbon Monoxide-validated 2-week point prevalence abstinence 4 weeks postquit date), determination to quit, age, ethnicity, Index of Multiple Deprivation (a summary score of 37 deprivation indicators for small geographical areas),^11^ gender (general sample only), previous prenatal smoking history and parity (pregnant sample only).

### Data Analysis

Response rates (%) with 95% confidence intervals (CIs) for the text assessments were computed and presented on graphs generated by Kaplan-Meier (KM) curve analyses. Repeated measure logistic regression for binary response within the framework of generalized estimating equation (GEE) was used to assess whether response rates changed significantly from the 3-week assessment to the subsequent assessments. GEE with the assumption of Poisson distribution and log link was used to assess whether the response time changed significantly from the 3-week assessment to the subsequent assessments.

Increases in response rate for each assessment after reminders were estimated with 95% CIs. The effect of the reminder on response time and ultimately response rate was assessed using the accelerated failure time model with the assumption of Weibull distribution. We bounded the maximum response time as 7 days after the original assessment message was sent.

Predictors of response to at least one smoking assessment text (excluding the self-efficacy assessment text) versus nonresponse were explored using bivariate logistic regression. Variables reaching a 5% significance level were entered simultaneously into a logistic regression model.

## Results

For the pregnant and general samples respectively, 99.0% and 98.0% had a white ethnic background, with median ages of 26 and 41, and 26.3% and 60.8% had high nicotine dependence (Table 1).

**Table 1.** Participant Characteristics

Characteristic	Pregnant smokers (*n* = 198) *n* (%)	General smokers (*n* = 293) *n* (%)
Female	198 (100)	155 (52.9)
Median age at enrolment (10th, 90th centile)	26 (19, 37)	41 (25, 61)
White	196 (99.0)	287 (98.0)
Determination to quit on 5-point scale (*SD*)	4.0 (1.0)	3.6 (0.6)
Number of cigarettes (p/d)		
1–5	58 (29.3)	4 (1.4)
6–10	78 (39.4)	61 (20.8)
11–20	58 (29.3)	165 (56.7)
≥21	4 (2.0)	63 (21.5)
Dependence category^a^		
Low	62 (31.3)	26 (8.9)
Medium	84 (42.4)	89 (30.6)
High	52 (26.3)	178 (60.8)
Index of multiple deprivation (*SD*)^b^	21.2 (15.7)	13.4 (8.1)

^a^Adapted from the Heaviness of Smoking Index. A dependence score was calculated by adding the scores of two items: cigarettes per day (1–5 = score of 0, 6–10 = 1, 11–20 = 2, 21–30 = 3, >30 = 4) and time to first cigarette after waking (>2h = 0, 1–2h = 1, 31–60min = 2, ≤30min = 3). A combined score of 0–2 = low dependence, 3–4 = medium dependence, 5–7 = high dependence.

^b^Index of Multiple Deprivation scores could not be calculated for four pregnant smokers and seven nonpregnant smokers. A higher score indicates greater deprivation.

### Response Rates to Text Message Assessments

**Figure 1.**
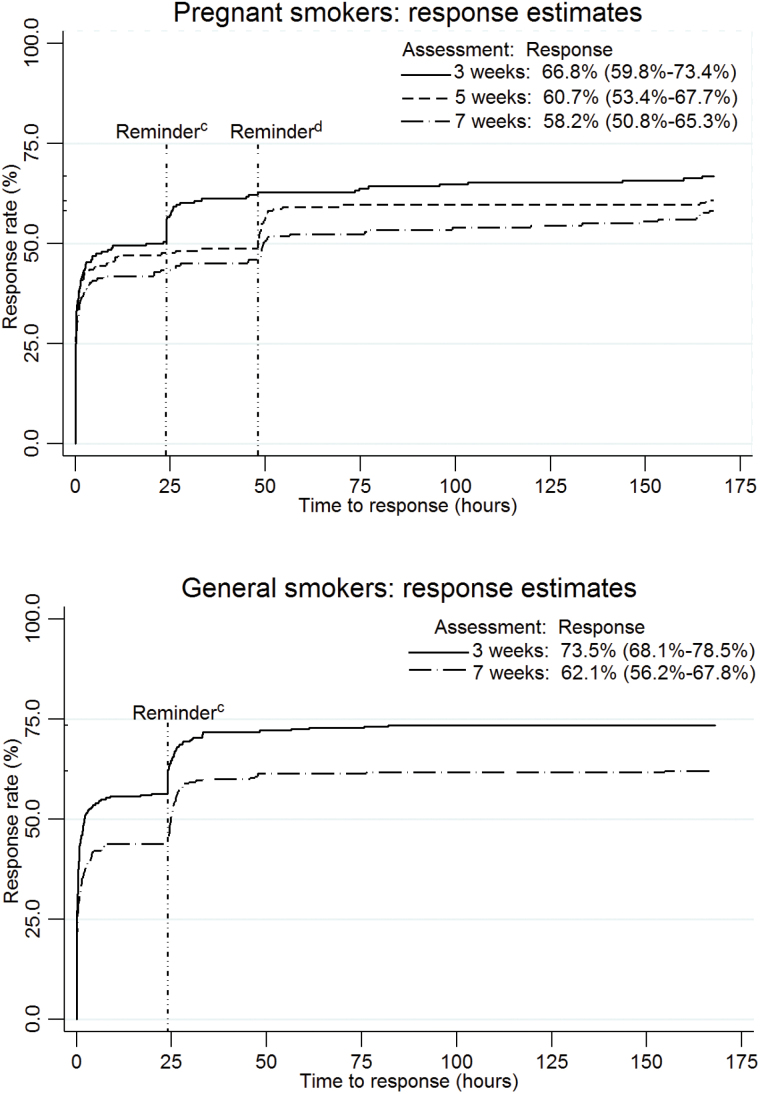
Kaplan-Meier curves by assessment for the probabilities of response after the assessments were sent (bounded at 168 hours/7 days)^a,b^. ^a^Four participants were missing from the 3-week assessments because there was no record of the time the assessment was sent (pregnant sample *n* = 2, general sample *n* = 2). Denominators reduced over time due to participant discontinuation of the program by texting STOP (pregnant sample *n* = 9, general sample *n* = 8). ^b^Eight pregnant participants who responded after 7 days were categorised as responding within 7 days. ^c^Reminder sent after 24 hours (pregnant sample 3-week assessment, general sample 3- and 7-week assessments). ^d^Reminder sent after 48 hours (pregnant sample 5- and 7-week assessments).

Among pregnant smokers, response rates to the assessments at 3, 5, and 7 weeks were 66.8% (95% CI 59.8–73.4), 60.7% (95% CI 53.4%–67.7%), and 58.2% (95% CI 50.8%–65.3%), respectively (Figure 1) with no significant differences between trial arms. Compared to 3-week response rates, the response at 5 weeks declined by 9.1% and at 7 weeks by 12.9%, but this was not statistically significant (GEE Wald test: χ^2^(2) = 3.22, *P* = .200). Among general smokers, 73.5% (95% CI 68.1%–78.5%) and 62.1% (95% CI 56.2%–67.8%) replied to the 3- and 7-week assessments, respectively. The response rate significantly declined from weeks 3 to 7 by 15.5% (GEE Wald test: χ^2^(1) = 8.56 *P* = .003).

### Time to Response to Text Message Assessments

Median response time in hours (interquartile range) for the pregnant sample was 0.31 (0.03–23.19), 0.33 (0.02–9.55), and 0.40 (0.02–26.37) at 3, 5, and 7 weeks, respectively with no significant differences between trial arms. For the general sample, it was 0.40 (0.04–7.68) and 0.88 (0.04–24.45) hours for the 3- and 7-week assessments. Increases in response times from the 3-week assessment to subsequent assessments were not statistically significant (pregnant: GEE Wald test: χ^2^(2) = 4.32, *P* = .115; general: GEE Wald test: χ^2^(1) = 1.65, *P* = .199).

### Impact of Reminders on Response

For pregnant smokers, after the reminders were sent, increases in response rate were 16.3% (95% CI 11.4%–22.3%), 12.0% (95% CI 7.8%–17.5%), and 13.2% (95% CI 8.7%–18.9%) at weeks 3, 5, and 7, respectively. Equivalent increases in response for general smokers were 17.2% (95% CI 13.0%–22.0%) and 18.2 % (95% CI 13.9%–23.2%) at weeks 3 and 7, respectively. The accelerated failure time model revealed a significant effect of the reminder on the response times for pregnant smokers (coefficient β = 4.61, 95% CI 4.08–5.13, *P* < .001) and general smokers (coefficient β = 4.00, 95% CI 3.62–4.38, *P* < .001).

### Predictors of Response to Assessment Messages

Among pregnant smokers, lower age (odds ratio [*OR*] = 0.95, 95% CI 0.91–1.00, *P* = .046) and lower IMD deprivation score (*OR* = 0.98, 95% CI 0.96–0.99, *P* = .011) were associated with response to a smoking assessment message. The strength of the associations changed little when these variables were entered into a multivariate model, although age no longer reached statistical significance (deprivation *OR* = 0.98, 95% CI 0.96–1.00, *P* = .013; age *OR* = 0.95, 95% CI 0.90–1.00, *P* = .053). Together these characteristics accounted for 7.6% of the variance in response. Among general smokers, only nonsmoking status at 4 weeks follow-up was associated with response (*OR* = 8.63, 95% CI 3.03–24.58, *P* < .001), accounting for 13.3% of variance.

## Discussion

This study provides a novel approach for examining the parameters of response to assessment text messages. Most smokers replying to an assessment text did so rapidly, though response rates reduced over time for general smokers, in line with a study of marijuana users.^12^ While response rates and timing were lower than that reported by others investigating smoking-related information collected by text message,^2,13^ the use of financial incentives^13^ and response training^2^ in these studies may explain such differences.

The current study is the first to formally assess the impact of text reminders on text assessments of health-related information. We found that reminders can significantly increase response, by 14% and 18% for pregnant and general smokers, respectively. The impact of reminders in our study was higher than that observed from a multicomponent response intervention that included four text reminders for children with anaemia.^14^ Our data supports the use of 24-hour reminders, with little benefit from extending them to 48 hours, and suggests nonresponse for a substantial minority may be due to being distracted or busy when receiving the initial message rather than disengagement. Sending further reminders to persistent nonresponders may increase response rate further, as found with postal questionnaires.^15^

Few participant characteristics predicted response, as found with email reminders for collecting general health information.^16^ Our findings suggest that older and more deprived pregnant smokers may be under-represented by text message collected data. Potential reasons for this include higher usage of text messaging among women aged 16–24 compared with older groups and the higher proportion of pre-pay mobiles, those susceptible to running out of “credit,” among those in the lowest compared to higher socioeconomic groups in the United Kingdom.^17^ The finding that smoking status predicted response among general smokers suggests that the commonly applied assumption in cessation studies that missing equals smoking^18^ may also be appropriate for simple and rapid digital data collection methods.

Study limitations include limited power for some secondary analyses, self-selection bias and observational data prone to confounding and bias. In addition, the smoking outcome predictor variable for pregnant smokers collected at 12 weeks follow-up was not close in time to the text assessments. While the text assessment frequency investigated may reflect real-world programmes, the data are probably less representative of high intensity assessments, such as Ecological Momentary Assessment.^2,19^ The generalizability of the findings to other populations and systems may be limited by contextual factors affecting response.

Given its low cost and speed of response, text messaging represents a useful tool for simple data collection from smokers over the short to medium term.

## Funding

The MiQuit feasibility trial was funded by Cancer Research UK (CR-UK) grant number C1345/A5809. The iQuit in Practice trial was funded by the National Institute for Health Research (NIHR) School for Primary Care Research (SPCR).

## Declaration of Interests

*None declared.*
